# Supplementary material for: Relationship of Different Anthropometric Indices with Vascular Ageing in an Adult Population without Cardiovascular Disease—EVA Study
Source: J Clin Med. 2022 May 9;11(9):2671. doi: 10.3390/jcm11092671 (PMC9105296; doi:10.3390/jcm11092671)
Supplement: Supplementary file 1 [file jcm-11-02671-s001.zip › jcm-1572698-supplementary.pdf]

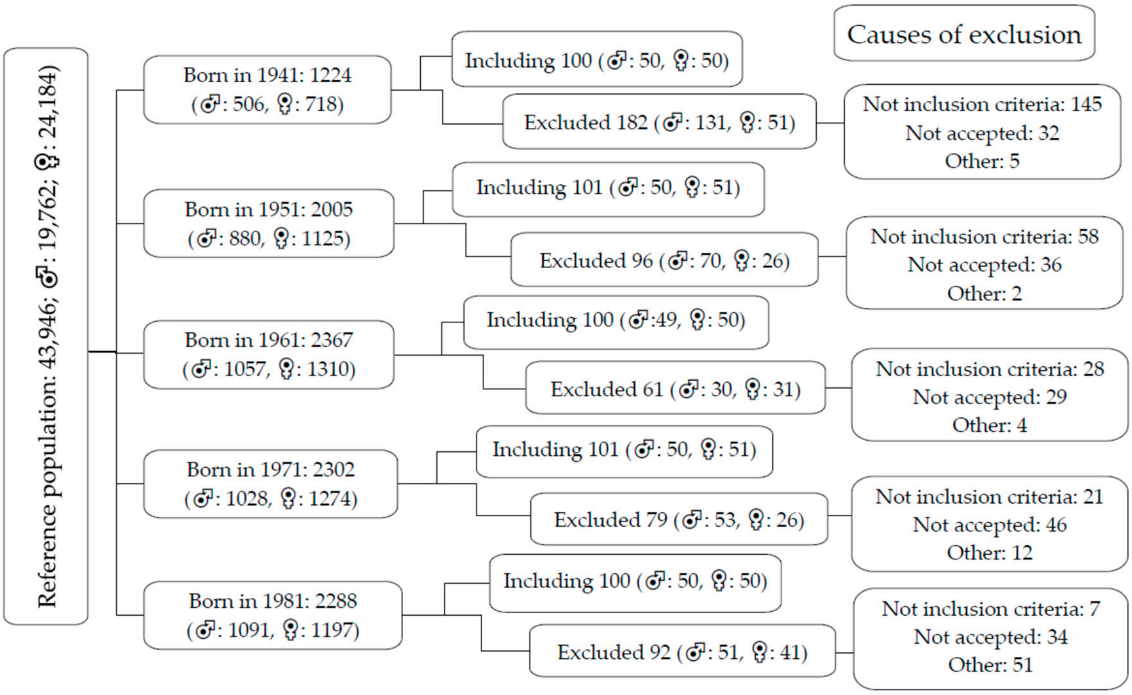

Figure S1. Flow diagram describing the reference population (43,946), those included and excluded, as well as exclusion criteria by age group and sex. A total of 259 subjects did not meet the inclusion criteria; 177 did not agree to participate in the study; 74 (other) subjects were not located due to changes in address or telephone number.

Table S1. Percentile values of the cf-PWV by age and sex.

**Age in year**	**35**	**45**	**55**	**65**	**75**
Men 90 th	8.00	8.40	9.20	12.70	15.80
Men 10 th	5.50	5.50	6.10	6.40	7.44
Women 90 th	7.30	7.88	9.38	9.62	13.30
Women 10 th	5.00	5.44	5.80	6.62	7,40

cf-PWV, carotid-femoral aortic pulse wave velocity.

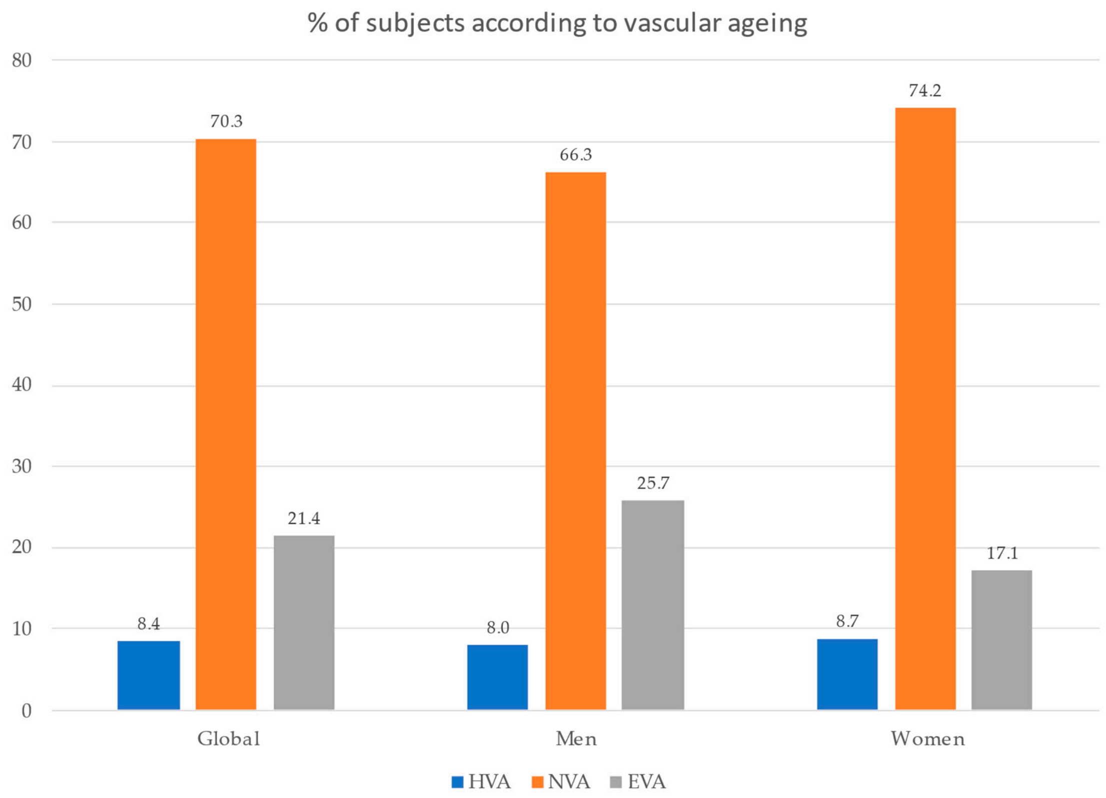

Figure S2. % Vascular ageing status global and by sex. EVA early vascular ageing, HVA healthy vascular ageing, NVA normal vascular ageing.

Table S2. Relationship between the anthropometric parameters with cf-PWV. Multiple regression analysis.

**Model 1**	B (95% CI)	P value
**BMI (kg/m²)**	0.137 (0.085 – 0.189)	<0.001
WC (cm)	0.076 (0.058 – 0.093)	<0.001
HC (cm)	0.022 (-0.002 – 0.047)	0.076
WHtR	0.014 (0.012 – 0.017)	<0.001
WHR	5.970 (4.208 – 7.734)	<0.001
BAI (%)	0.059 (0.022 – 0.096)	0.002
VAI (cm²)	0.196 (0.103 – 0.288)	<0.001
BRI	0.674 (0.543 – 0.805)	<0.001
CUNBAE	0.057 (0.029 – 0.085)	<0.001
AVI	0.208 (0.161 – 0.256)	<0.001
SATA	0.006 (0.004 – 0.008)	<0.001
IMP	0.032 (0.021 – 0.044)	<0.001
Model 2		
BMI (kg/m²)	0.063 (0.022 – 0.103)	0.002
WC (cm)	0.036 (0.020 – 0.052)	<0.001
HC (cm)	0.026 (0.008 – 0.044)	0.004
WHtR	0.005 (0.002 – 0.008)	<0.001
WHR	0.958 (-0.707 – 2.622)	0.259
BAI (%)	0.032 (-0.002 – 0.066)	0.064
VAI (cm²)	0.118 (0049 – 0.187)	0.001
BRI	0.251 (0.133 – 0.369)	<0.001
CUNBAE	0.048 (0.015 – 0.081)	0.005
AVI	0.101 (0.058 – 0.144	<0.001
SATA	0.003 (0.001 – 0.004)	0.002
IMP	0.013 (0.004 – 0.022)	0.006
Model 3		
BMI (kg/m²)	0.043 (0.004 – 0.081)	0.032
WC (cm)	0.027 (0.012 – 0.043)	0.001
HC (cm)	0.021 (0.004 – 0.038)	0.018
WHtR	0.003 (0.001 – 0.006)	0.006
WHR	0.606 (-0.956 – 2.169)	0.446
BAI (%)	0.606 (-0.956 – 2.169)	0.446
VAI (cm²)	0.018 (-0.015 – 0.050)	0.285
BRI	0.098 (0.024 – 0.172)	0.010
CUNBAE	0.174 (0.059 – 0.288)	0.003
AVI	0.033 (0.002 – 0.065)	0.040
SATA	0.078 (0.037 – 0.119)	<001
IMP	0.002 (001 – 0.004)	0.032

Multiple regression analysis using cf-PWV dependent variables, anthopemetric indices as independent variable. Model 1 unadjusted. Model 2 adjusted by age in years, sex (Male = 1 and female = 0). Model 3 adjusted by age in years, sex CVRF (dyslipidemia, tobacco use, hypertension and diabetes mellitus type 2) and IV (0 = absence, 1 = presence). CVRF, cardiovascular risk factors; IV, vascular injury; BMI, Body mass index; WC, Waist circumference; HC, Hip circumference; WHtR, waist-to-height ratio; WHR, waist-to-hip ratio; BAI, body adiposity index; VAI, Visceral adiposity index; BRI, body roundness index; AVI, Abdominal Volume Index; CUNBAE, Clinica Universidad de Navarra body adiposity estimator; SATA, Subcutaneous Adipose Tissue Area; IMP, Ideal Mass Percentage.

Table S3. AUCs, optimal cut-off, sensitivity, specificity, for the anthropometric indices in ROC analysis for predicting healthy vascular ageing.

Anthropometric Indices	AUC (95% CI)	p	Cut-off	Sensitivity	Specificity	Youden Index
**Global**						
BMI	0.72 (0.65-0.80)	<0.001	24	0.74	0.60	0.34
SATA	0.72 (0.66-0.80)	<0.001	229	0.74	0.60	0.34
IMP	0.72 (0.64-0.80)	<0.001	103	0.71	0.69	0.40
WHtR	0.67 (0.60-0.75)	<0.001	0.54	0.66	0.63	0.29
BRI	0.67 (0.60-0.75)	<0.001	4	0.66	0.63	0.29
BAI	0.63 (0.55-0.71)	0.006	29	0.60	0.65	0.25
WC	0.67 (0.60-0.74)	<0.001	90	0.60	0.60	0.20
AVI	0.67 (0.53-0.74)	<0.001	16	0.61	0.60	0.21
HC	0.66 (0.58-0.75)	<0.001	100	0.63	0.67	0.30
CUNBAE'	0.65 (0.57-0.74)	0.001	28	0.75	0.52	0.27
VAI	0.61 (0.53-0.69)	0.018	9	0.61	0.62	0.22
WHR	0.60 (0.53-0.68)	0.025	89	0.58	0.60	0.18
**Men**						
BMI	0.71 (0.60-0.82)	0.002	25	0.72	0.70	0.42
SATA	0.71 (0.64-0.84)	0.002		253	0.71	0.70
IMP	0.71 (0.59-0.82)	0.002	105	0.66	0.75	0.41
WHtR	0.69 (0.60-0.78)	0.005	0.56	0.60	0.75	0.35
BRI	0.69 (0.60-0.78)	0.005	4.5	0.60	0.75	0.35
BAI	0.59 (0.46-0.71)	0.206	27	0.61	0.60	0.21
WC	0.73 (0.64-0.81)	0.001	96	0.62	0.80	0.42
AVI	0.72 (0.63-0.81)	0.001	19	0.61	0.85	0.46
HC	0.71 (0.52-0.75)	0.002	100	0.65	0.65	0.30
CUNBAE'	0.71 (0.61-0.82)	0.002	27	0.63	0.80	0.43
VAI	0.56 (0.55-0.74)	0.353	9	0.65	0.58	0.23
WHR	0.68 (0.56-0.80)	0.007	0.83	0.56	0.64	0.20
**Women**						
BMI	0.74 (0.64-0.84)	<0.001	24	0.68	0.78	0.46
SATA	0.74 (0.64-0.84)	<0.001	227	0.68	0.78	0.46
IMP	0.74 (0.63-0.84)	<0.001	103	0.68	0.78	0.46
WHtR	0.67 (0.55-0.77)	0.013	0.53	0.59	0.78	0.37
BRI	0.67 (0.55-0.77)	0.013	4	0.59	0.78	0.37
BAI	0.70 (0.58-0.72)	0.002	29	0.79	0.60	0.39
WC	0.64 (0.53-0.75)	0.029	84	0.62	0.64	0.26
AVI	0.65 (0.54-0.76)	0.023	14	0.67	0.60	0.27
HC	0.69 (0.57-0.80)	0.004	99	0.67	0.69	0.36
CUNBAE'	0.73 (0.62-0.83)	<0.001	37	0.65	0.82	0.47
VAI	0.65 (0.55-0.74)	0.023	9	0.61	0.69	0.30
WHR	0.56 (0.45-0.67)	0.353	0,92	0.67	0.70	0.37

AUC, area under curve; BMI, Body mass index; WC, Waist circumference; HC, Hip circumference; WHtR, waist-to-height ratio; WHR, waist-to-hip ratio; BAI, body adiposity index; VAI, Visceral adiposity index; BRI, body roundness index; AVI, Abdominal Volume Index; CUN-BAE, Clinica Universidad de Navarra body adiposity estimator; SATA, Subcutaneous Adipose Tissue Area; IMP, Ideal Mass Percentage.

Table S4. AUCs, optimal cut-off, sensitivity, specificity, for the anthropometric indices in ROC analysis for predicting early vascular ageing.

Anthropometric Indices	AUC (95% CI)	p	Cut-off	Sensitivity	Specificity	Youden Index
**Global**						
BMI	0.56 (0.50-0.62)	0.052	26	0.63	0.53	0.16
SATA	0.56 (0.50-0.62)	0.052	274	0.63	0.53	0.16
IMP	0.55 (0.49-0.62)	0.084	109	0.60	0.53	0.13
WHtR	0.60 (0.54-0.66)	0.002	0.57	0.62	0.59	0.21
BRI	0.60 (0.54-0.66)	0.002	4.8	0.62	0.59	0.21
BAI	0.51 (0.44-0.57)	0.883	29	0.60	0.48	0.08
WC	0.60 (0.54-0.66)	0.001	91	0.68	0.52	0.20
AVI	0.60 (0.54-0.66)	0.001	17	0.68	0.50	0.18
HC	0.55 (0.49-0.61)	0.099	92	0.54	0.62	0.18
CUNBAE'	0.50 (0.44-0.56)	0.976	32	0.51	0.49	0.00
VAI	0.58 (0.52-0.64)	0.011	10	0.64	0.50	0.14
WHR	0.60 (0.54-0.66)	0.002	94	0.53	0.63	0.16
**Men**						
BMI	0.53 (0.45-0.61)	0.465	26	0.62	0.46	0.08
SATA	0.53 (0.45-0.61)	0.465	275	0.62	0.46	0.08
IMP	0.54 (0.46-0.62)	0.354	108	0.63	0.51	0.14
WHtR	0.59 (0.51-0.67)	0.035	0.58	0.61	0.58	0.20
BRI	0.59 (0.51-0.67)	0.035	4.95	0.61	0.58	0.20
BAI	0.56 (0.48-0.64)	0.178	28	0.58	0.62	0.20
WC	0.58 (0.50-0.66)	0.052	98	0.61	0.54	0.15
AVI	0.58 (0.50-0.66)	0.062	19	0.61	0.54	0.15
HC	0.54 (0.46-0.62)	0.348	102	0.61	0.54	0.15
CUNBAE'	0.58 (0.50-0.66)	0.069	28	0.60	0.56	0.16
VAI	0.59 (0.50-0.67)	0.041	13	0.56	0.60	0.16
WHR	0.58 (0.50-0.65)	0.070	0.96	0.61	0.59	0.20
**Women**						
BMI	0.57 (0.47-0.67)	0.136	26	0.60	0.62	0.22
SATA	0.57 (0.47-0.67)	0.136	278	0.60	0.62	0.22
IMP	0.58 (0.47-0.68)	0.117	113	0.60	0.60	0.20
WHtR	0.58 (0.48-0.68)	0.092	0.57	0.58	0.64	0.22
BRI	0.58 (0.48-0.68)	0.092	4.85	0.58	0.64	0.22
BAI	0.58 (0.47-0.68)	0.119	35	0.53	0.62	0.15
WC	0.57 (0.47-0.67)	0.132	88	0.63	0.58	0.21
AVI	0.57 (0.47-0.67)	0.136	16	0.62	0.60	0.22
HC	0.57 (0.47-0.66)	0.165	102	0.60	0.51	0.11
CUNBAE'	0.58 (0.48-0.68)	0.095	39	0.63	0.54	0.17
VAI	0.56 (0.46-0.66)	0.194	11	0.56	0.60	0.16
WHR	0.55 (0.46-0.64)	0.271	0.84	0.67	0.51	0.18

AUC, area under curve; BMI, Body mass index; WC, Waist circumference; HC, Hip circumference; WHtR, waist-to-height ratio; WHR, waist-to-hip ratio; BAI, body adiposity index; VAI, Visceral adiposity index; BRI, body roundness index; AVI, Abdominal Volume Index; CUN-BAE, Clinica Universidad de Navarra body adiposity estimator; SATA, Subcutaneous Adipose Tissue Area; IMP, Ideal Mass Percentage.
